# Supplementary material for: Vaccine Based on Dendritic Cells Electroporated with an Exogenous Ovalbumin Protein and Pulsed with Invariant Natural Killer T Cell Ligands Effectively Induces Antigen-Specific Antitumor Immunity
Source: Cancers (Basel). 2021 Dec 30;14(1):171. doi: 10.3390/cancers14010171 (PMC8750915; doi:10.3390/cancers14010171)
Supplement: Supplementary file 1 [file cancers-14-00171-s001.zip › cancers-1524696-supplementary.pdf]

Supplementary Materials

Vaccine Based on Dendritic Cells Electroporated with an Exogenous Ovalbumin Protein and Pulsed with Invariant Natural Killer T Cell Ligands Effectively Induces Antigen-Specific Antitumor Immunity

Akihiro Watanabe, Kimihiro Yamashita, Mitsugu Fujita, Akira Arimoto, Masayasu Nishi, Shiki Takamura, Masafumi Saito, Kota Yamada, Kyosuke Agawa, Tomosuke Mukoyama, Masayuki Ando, Shingo Kanaji, Takeru Matsuda, Taro Oshikiri and Yoshihiro Kakeji

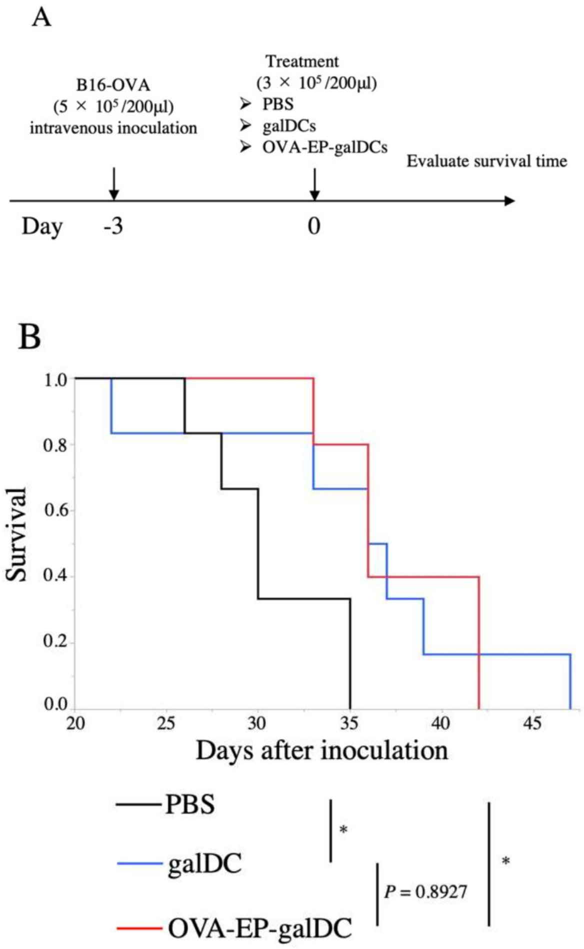

Figure S1. OVA-EP-galDCs as well as galDCs prolonged the survival of tumor-bearing mice in B16F10 experimental lung metastases models. (**A**) B16-OVA tumor cells were intravenously injected to C57BL/6 mice (Day -3). Three days after tumor cell injection (Day 0), the mice were treated with PBS, galDCs, or OVA-EP-galDCs (5×10^5 cells/mouse) (**B**) Survival analysis of B16F10 experimental lung metastases models treated with PBS, galDCs, or OVA-EP-galDCs was conducted ($n = 5$). To analyze survival times, Kaplan-Meier test was performed. *$p < 0.05$.

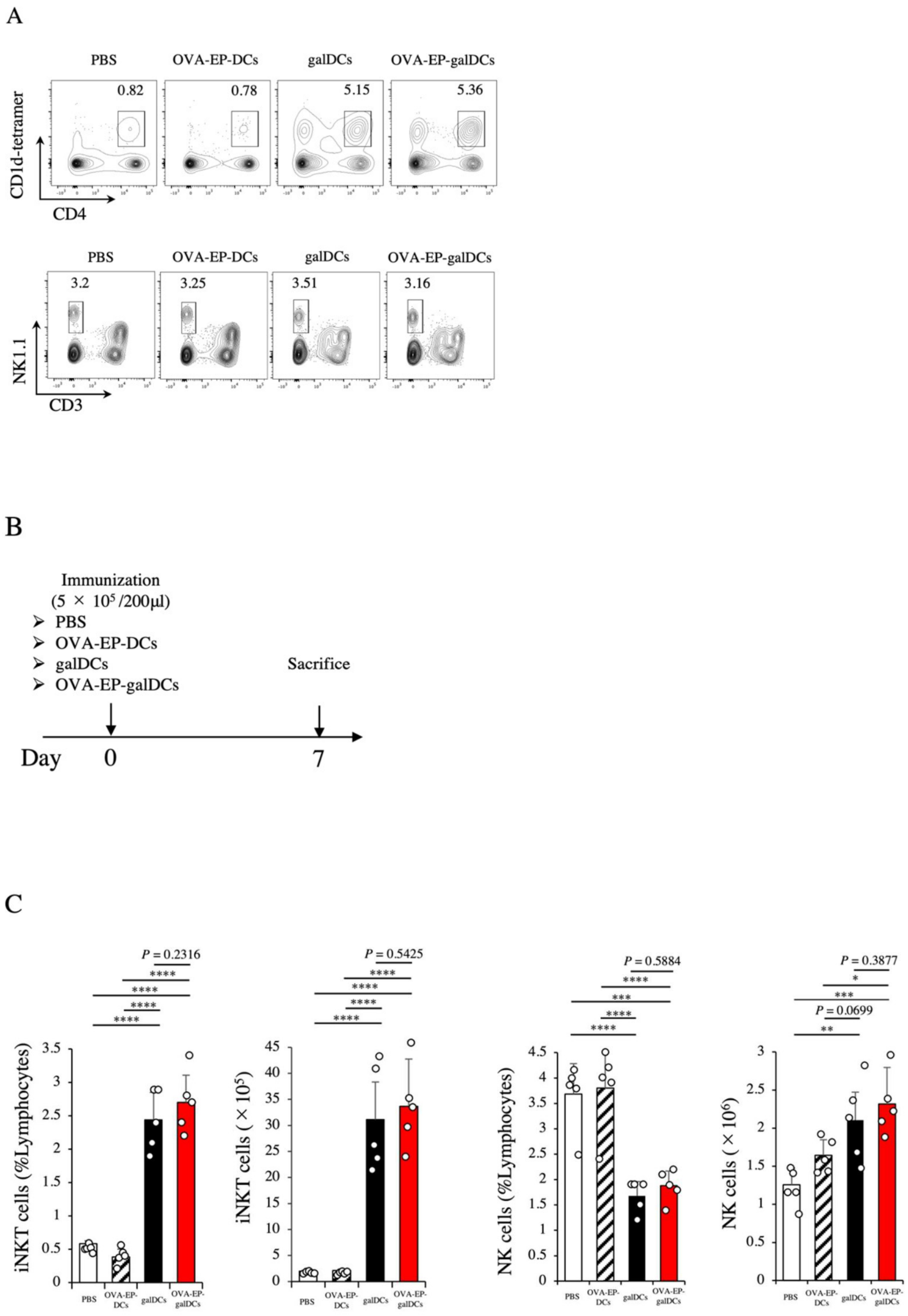

A
PBS
OVA-EP-DCs
galDCs
OVA-EP-galDCs
0.82
0.78
5.15
5.36
CD1d-tetramer
CD4
3.2
3.25
3.51
3.16
NK1.1
CD3
B
Immunization
(5 × 10⁵ /200μl)
PBS
OVA-EP-DCs
galDCs
OVA-EP-galDCs
Sacrifice
Day
0
7
C
iNKT cells (%Lymphocytes)
P = 0.2316
iNKT cells (× 10⁵)
P = 0.5425
NK cells (%Lymphocytes)
P = 0.5884
NK cells (× 10⁶)
P = 0.3877
P = 0.0699

D

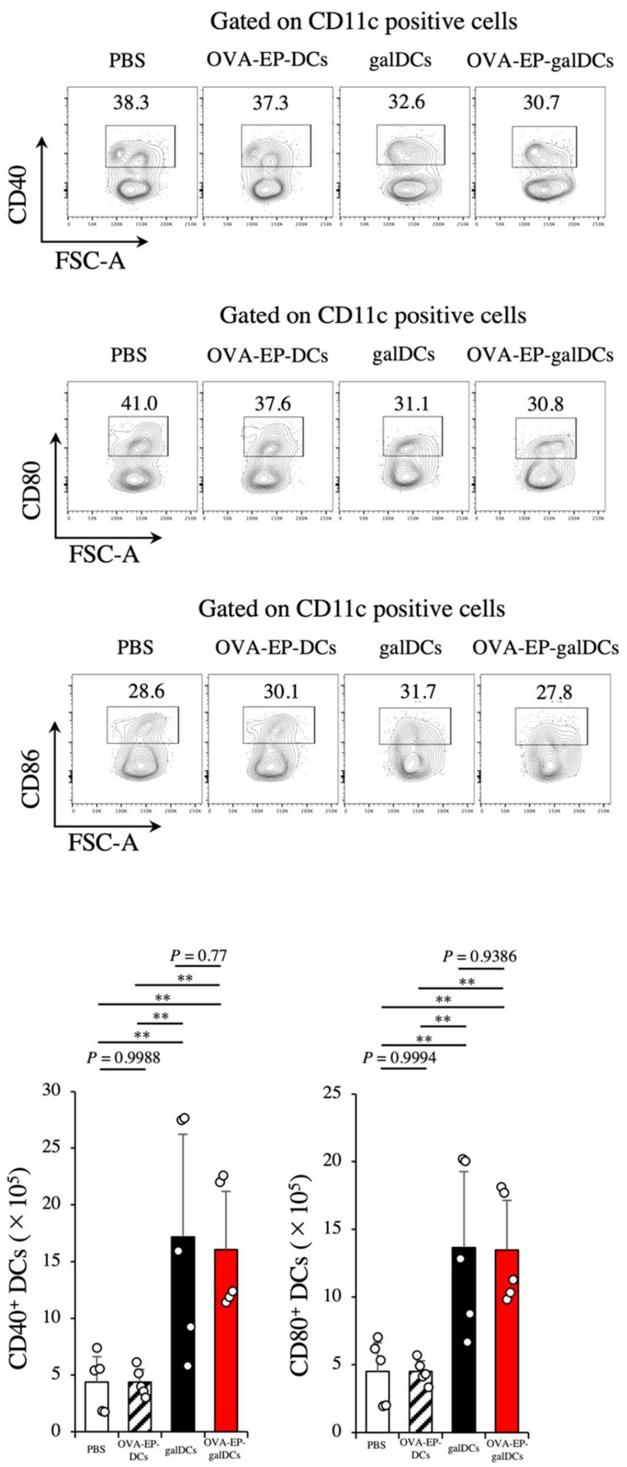

Figure S2. OVA-EP-galDCs activated iNKT cells, NK cells, and DCs in spleen. (**A**) Typical examples of flow cytometric analysis of iNKT cells and NK cells on Day 4. (**B**) Experimental schema is shown. C57BL/6 mice were immunized with PBS, galDCs, or OVA-EP-galDCs (5×10^5 cells/mouse). On Day 7 after the immunization, the frequency and the number of iNKT cells and NK cells in spleen were assessed by flow cytometry. (**C**) The frequency and the number of iNKT cells and NK cells on Day 7 ($n = 5$). (**D**) Typical examples of flow cytometric analysis of activated DCs in spleen and the number of $CD40^+$ DCs and $CD80^+$ DCs on Day 4 were shown ($n = 5$). To analyze statistical differences among multiple groups, ANOVA with Holm's post-hoc test was performed. *$p < 0.05$, **$p < 0.01$, ***$p < 0.001$, and ****$p < 0.0001$.
